# Automated integrative high-throughput phenotyping of plant shoots: a case study of the cold-tolerance of pea (*Pisum sativum* L.)

**DOI:** 10.1186/s13007-015-0063-9

**Published:** 2015-03-19

**Authors:** Jan F Humplík, Dušan Lazár, Tomáš Fürst, Alexandra Husičková, Miroslav Hýbl, Lukáš Spíchal

**Affiliations:** Department of Chemical Biology and Genetics, Centre of the Region Haná for Biotechnological and Agricultural Research, Faculty of Science, Palacký University, Šlechtitelů 11, Olomouc, CZ-78371 Czech Republic; Department of Biophysics, Centre of the Region Haná for Biotechnological and Agricultural Research, Faculty of Science, Palacký University, Šlechtitelů 11, Olomouc, CZ-78371 Czech Republic; Department of Mathematical Analysis and Applications of Mathematics, Faculty of Science, Palacký University, 17. listopadu 12, Olomouc, CZ-77146 Czech Republic; Department of Genetic Resources for Vegetables, Medicinal and Special Plants, Centre of the Region Haná for Biotechnological and Agricultural Research, Crop Research Institute, Šlechtitelů 11, Olomouc, CZ-78371 Czech Republic

**Keywords:** Plant phenotyping, RGB digital imaging, Chlorophyll fluorescence imaging, Shoot growth, Biomass production, Cold adaptation, Pea (*Pisum)*

## Abstract

**Background:**

Recently emerging approaches to high-throughput plant phenotyping have discovered their importance as tools in unravelling the complex questions of plant growth, development and response to the environment, both in basic and applied science. High-throughput methods have been also used to study plant responses to various types of biotic and abiotic stresses (drought, heat, salinity, nutrient-starving, UV light) but only rarely to cold tolerance.

**Results:**

We present here an experimental procedure of integrative high-throughput in-house phenotyping of plant shoots employing automated simultaneous analyses of shoot biomass and photosystem II efficiency to study the cold tolerance of pea (*Pisum sativum* L.). For this purpose, we developed new software for automatic RGB image analysis, evaluated various parameters of chlorophyll fluorescence obtained from kinetic chlorophyll fluorescence imaging, and performed an experiment in which the growth and photosynthetic activity of two different pea cultivars were followed during cold acclimation. The data obtained from the automated RGB imaging were validated through correlation of pixel based shoot area with measurement of the shoot fresh weight. Further, data obtained from automated chlorophyll fluorescence imaging analysis were compared with chlorophyll fluorescence parameters measured by a non-imaging chlorophyll fluorometer. In both cases, high correlation was obtained, confirming the reliability of the procedure described.

**Conclusions:**

This study of the response of two pea cultivars to cold stress confirmed that our procedure may have important application, not only for selection of cold-sensitive/tolerant varieties of pea, but also for studies of plant cold-response strategies in general. The approach, provides a very broad tool for the morphological and physiological selection of parameters which correspond to shoot growth and the efficiency of photosystem II, and is thus applicable in studies of various plant species and crops.

## Introduction

In plants, acclimation to cold, causes reduced growth, increase in antioxidant content, reduced water content, and changes in gene regulation, hormone balance, membrane composition, osmotic regulation, and photosynthetic function [1]. The adaptability and productivity of legumes (chickpea, faba bean, lentil, and pea) are limited by abiotic stresses in general [2], and their high sensitivity to chilling and freezing temperatures is well described [3].

Since cold tolerance is an important agronomical problem in Central and Northern Europe and geographically similar regions, we aimed to develop a routine measuring procedure for automated integrative high-throughput screening for selection of potentially cold tolerant cultivars. Pea (*Pisum sativum* L.) was chosen as a model crop because its tolerance to cold stress is one of the limiting factors in autumn sowings which allows for the enhanced productivity of pea plants. Overwintering plants have developed adaptive responses to seasonal weather changes. For example, overwintering evergreens have developed so-called sustained non-photochemical quenching (reviewed, e.g., by Verhoeven [4]) as a protection mechanism against absorbed light which is in excess with respect to the capacity of the carbon photosynthetic reactions and which is decreased during winter. These plants sense the upcoming cold period through the perception of environmental impulses, mainly temperature and day length. However, the sustained non-photochemical quenching does not work in modern pea cultivars. For this reason, we chose two modern cultivars and investigated their reaction to cold stress. We employed digital RGB imaging to study shoot growth, and chlorophyll (Chl) fluorescence imaging (CFIM) to analyze various parameters of plant photosystem II (PSII) efficiency. The cultivars used in this study were morphologically similar which facilitated the validation of sensitivity and resolution of our visible imaging analysis.

There is a paucity of information on the acclimation of pea plants to cold. An extensive study was published by Markarian et al. [5]. These authors evaluated 26 pea lines based on their winter survival. Further physiological parameters (total dry matter and photosynthetic area) of autumn- and spring-sown pea plants were evaluated by Silim et al. [6]. Autumn-sown plants produced similar seed yields to spring sowings when the winter survival was adequate, and autumn sowings matured 2–4 weeks before the spring-sown crops, depending on the variety and season [6]. The effects of short term acclimation (four days) of pea plants to cold temperatures (5°C) were explored by Yordanov et al. [7] who measured the rate of oxygen production and CO_2_ assimilation, and Chl fluorescence parameters in order to evaluate photochemical activity and functional heterogeneity of PSII. They found that cold-acclimated plants showed higher photosynthetic rates and better Chl fluorescence parameters than non-acclimated plants [7]. The effects of short term cold acclimation (three days, 4°C) and subsequent recovery (2 days) of standard pea plants were studied by Chl fluorescence measurements in more detail by Georgieva and Lichtenthaler [8]. The Chl fluorescence parameters reflecting photosynthetic function decreased during cold acclimation but were reversible in the subsequent recovery [8]. A similar study was later carried out with three different pea cultivars by Georgieva and Lichtenthaler [9].

These studies revealed the importance of two potential traits that could be used to distinguish between pea cultivars with different cold-sensitivity: rate of shoot growth and values of Chl fluorescence parameters. Both traits can now be studied by non-invasive high-throughput platforms to provide integrative insight into plant physiology during cold acclimation. The spatio-temporal changes in shoot biomass or leaf area can be assessed using automated RGB imaging and image-analysis software, as has been shown for many species such as cereals, tomatoes, soybean and beans [10-13]. The Chl fluorescence parameters are routinely analyzed by non-imaging fluorometres (NICF) or the imaging system (CFIM). For physiological studies, kinetic types of CFIM that allow computation of various Chl fluorescence parameters on the whole leaf or shoot are the most valuable. However, the kinetic type CFIM has not been commonly integrated into high-throughput systems [14] and in recent reports only systems measuring a single Chl fluorescence level have been employed [11,15]. The intensity of Chl fluorescence depends on the amount of chlorophylls; thus, a single Chl fluorescence level can be used, e.g., to distinguish between non-stressed and senescent leaves (when amount of Chls is decreased) at late stages of stress. However, this does not provide any information about earlier processes in PSII that are not necessarily linked to later senescence events.

In this report, we describe a procedure employing an automated integrative high-throughput platform suitable for studies of the physiological basis of cold-stress adaptation and selection of pea cultivars with cold sensitivity/tolerance. The platform measures shoot area and Chl fluorescence to provide a complex analysis of plants during cold-acclimation. For this purpose, we developed new software for automatic RGB image analysis and we evaluated various parameters of Chl fluorescence obtained from CFIM. The data from the automated phenotyping platform were validated through estimation of shoot biomass by manual weighing of the shoots and by measurement of Chl fluorescence by a NICF hand operated fluorometer. Despite the complexity of pea shoots, very good correlation between pixel based shoot area and fresh biomass were obtained. Similarly, the Chl fluorescence parameters measured by NICF fully confirmed the reliability of the automated CFIM analysis.

## Results and discussion

### Visible imaging used for shoot growth

**Figure 1:**
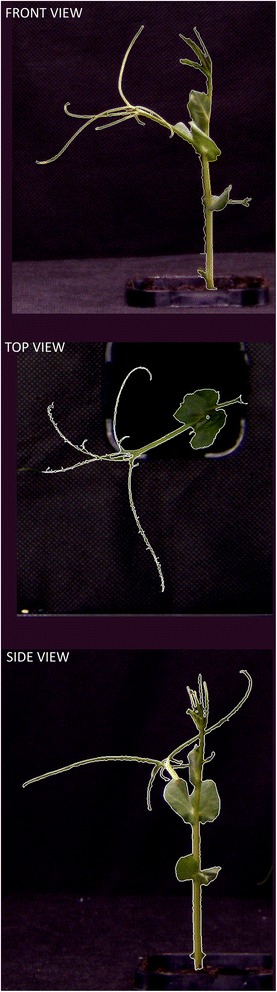
**The example images of three optical projections of single END seedling used for calculation of total green area on 8th day of cold acclimation.** The green area that was digitally extracted from the images is marked by white border line.

**Figure 2:**
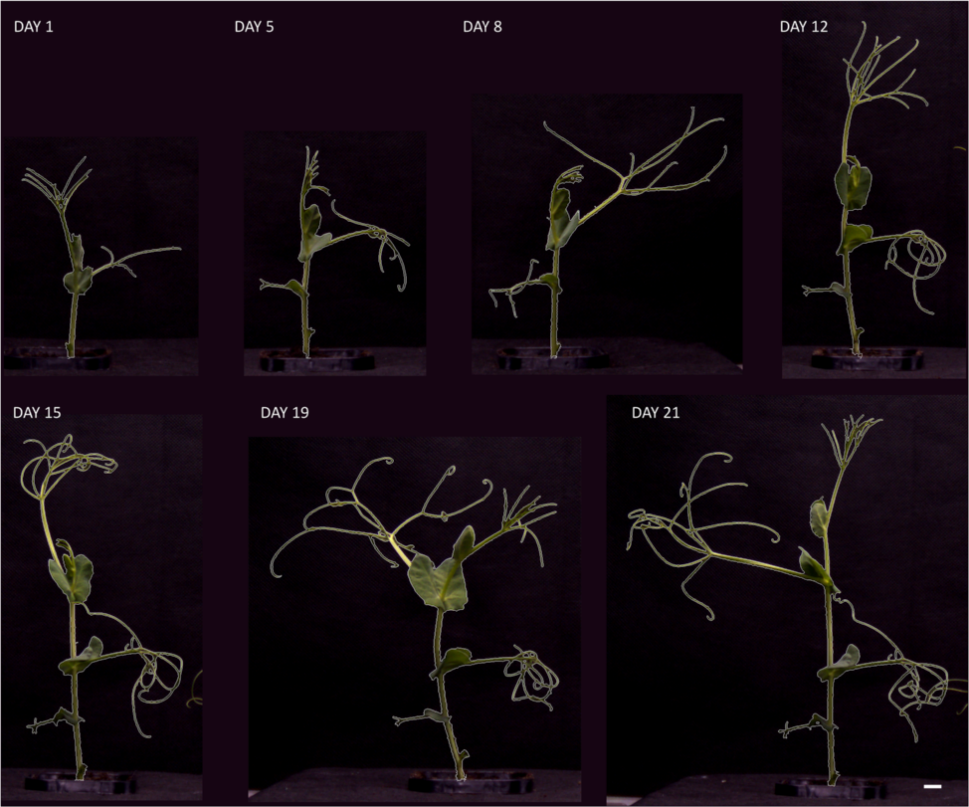
**Example images taken from front view camera showing the growth progress of the TER seedling during cold acclimation for 21 days.** The green area that was digitally extracted from the images is marked by white border line. The white bar in the right bottom site represents length of 1 cm.

**Figure 3:**
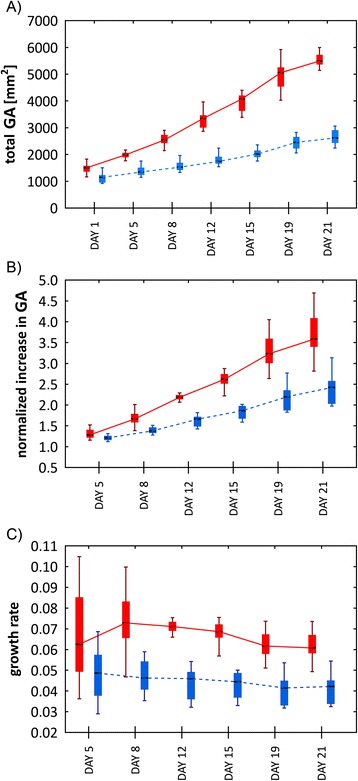
**Analyses of the growth progress of shoots of TER (red boxes – full line) and END (blue boxes – dashed line) pea cultivars.** The values derived from the green area on n^th^ days (1, 5, 8,…, 21) are presented as medians (black bars) and quartiles (boxes). For better readability, the boxes are shifted in x-axes to not to overlap, but still represent the values measured on the same days. **A)** A total green area. **B)** A normalized green area. **C)** A relative growth rate. The error bars show minimal and maximal values.

To compare the influence of cold acclimation on biomass production, two putative cold-resistant cultivars of pea Terno and Enduro were selected (labeled as TER and END, respectively). After germination, the seedlings were grown in a growth chamber at 22/20°C (see Materials and methods) and after the development of the first true leaf, the cold stress conditions were established. The seedlings continued growing in 5°C for 21 days and were screened twice per week in the automated platform. The green area of each individual seedling was extracted from particular projections (Figure 1) and combined to account for the overall shoot biomass. As shown in Figure 2, the total green area of the plants was calculated at 7 time-points. The cultivar TER showed a significantly higher (for p values see Table 1) increase in the total green area compared to the cultivar END (Figure 3A). Because the green area of the cultivars was different at the beginning of the experiment, the normalized green area (NGA) was calculated, where the green area on the n^th^ (5, 8, … 21) day of measurement was divided by the green area obtained on the 1st measuring day. The TER cultivar showed higher shoot growth which on the 21st day was almost a 3.5 fold increase in the green area, whereas END multiplied its projected area by only about 2.5-times (Figure 3B). To analyze how the cultivars differed in their growth rates, the relative growth rate (RGR) was used according to Hoffmann and Poorter [16]. We used the following formula:

**Table 1:** **The p values of the Mann–Whitney test of statistical significant difference of growth parameters based on RGB imaging**

**Measuring day**	**Green area p value**	**NGA p value**	**Growth rate p value**
1	<0.001	n. a.	n. a.
5	<0.001	<0.003	<0.003
8	<0.001	<0.001	<0.001
12	<0.001	<0.001	<0.001
15	<0.001	<0.001	<0.001
19	<0.001	<0.001	<0.001
21	<0.001	< 0.001	< 0.001

For each day the comparisons between TER and END datasets were tested in all three parameters respectively.

$$ RGR=\frac{\overline{ln{W}_2}-\overline{ln{W}_1}}{t_2-{t}_1} $$

where $\overline{ln{W}_1}$ and $\overline{ln{W}_2}$ are the means of the natural logarithms of the plant’s green areas and t_1_ and t_2_ are the times at which the green areas were measured. The TER cultivar relative growth rate was significantly higher (for p values see Table 1) during the whole period of cold acclimation. Moreover, at the beginning of the cold stress, the TER cultivar tended to speed-up its growth, then reached a steady state and finally decreased its RGR by the end of the experiment. The second cultivar, END, was very stable, slightly decreasing its growth rate during the experiment (Figure 3C). To examine the statistical significance of the differences between obtained TER and END growth-related parameters, the non-parametric Mann–Whitney *U* test was performed for each measuring day. The p values obtained for each measuring day are shown in Table 1.

**Figure 4:**
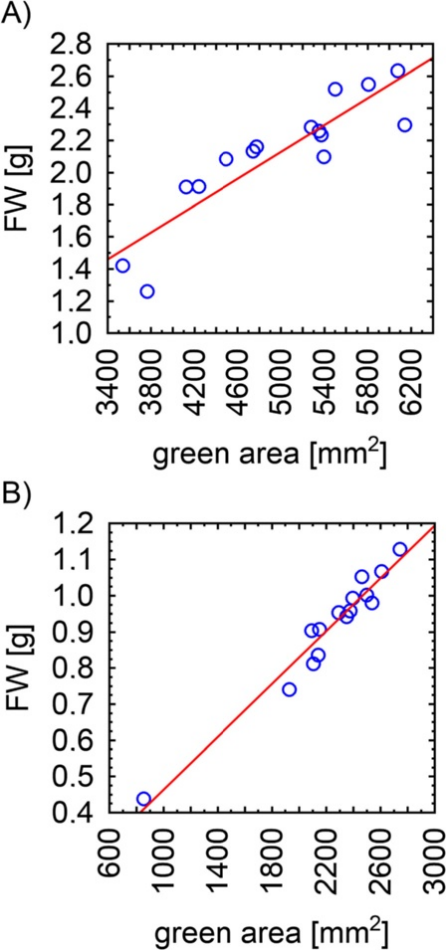
**The correlation of the green area and biomass.** The Spearmann correlation coefficients of FW and green area of TER cultivar **A)** and END cultivar **B)** were 0.91 and 0.96, respectively (p value < 0.05).

It has been reported that cold-treatment affects total shoot biomass production and growth-rate in spring-sown and overwintering pea cultivars [6,17]. Besides shoot growth cold-treatment affects also growth of the root as showed in work by Bourion et al. [17]. However, the effect on the root is less severe compared to the above ground parts of the plants [17]. Due to this fact and due to the technical set up of our automated platform in this study we focused only on the analyses of cold-treatment effects on shoot growth. We describe here the development of the measuring setup for automated screening of pea cultivars with different cold-sensitivity through analysis of the shoot growth by RGB imaging followed by precise image-analysis. A similar approach has been shown for different species and different types of stresses. Considering crop species alone, most of the protocols for automated phenotyping using RGB imaging were designed for cereals, most often to screen for drought, or salt tolerant plants [10,15,18-23]. Surprisingly, use of such a method has not been presented so far for any crops studied for cold-acclimation. Although there was no presumed effect of cold-treatment on the reliability of RGB imaging, the complicated morphology of field pea cultivars could potentially affect the accuracy of the automated measurements. For this reason, we tested our method of the green area (or projected area) estimation from automated RGB imaging by its comparison with a method of manual weighing of the shoots. The shoots of both cultivars were harvested on the last measuring day and FW of individual plant shoots was measured. Subsequently, correlations between the green area and FW were calculated using the non-parametric Spearman correlation coefficient. A similar approach has been reported recently by Hairmansis et al. [15] for rice. These authors found a correlation of projected area and FW ranging from 0.96 to 0.97. A more sophisticated calculation was developed by Golzarian et al. [22] who used estimated shoot area as a function of plant area and plant age. This method was applied by Pereyra-Irujo et al. [12] in experiments with soybean, providing a correlation of 0.97 in dry mass. Shoots of cereals and soybean have relatively low spatial-complexity. In contrast, shoots of field pea cultivars TER and END are formed mainly by stem and tiny tendrils (Figures 1, 2) requiring very precise identification by image-analysis software. Despite the challenging pea shoot morphology, Spearman correlation coefficients of 0.91 and 0.96 for TER and END cultivars, respectively, were found in our analysis (p < 0.05; Figure 4). This is fully comparable with the phenotyping protocols designed for other crop species and provides an efficient and reliable tool for the evaluation of pea growth.

### Chlorophyll fluorescence imaging used for determination of photosynthetic function

Further variables used for phenotyping of the two pea cultivars were those obtained from measurements of Chl fluorescence induction (CFIN), which reflects photosynthetic function, mainly of PSII. Based on our knowledge of the parameters that can be determined from CFIN (reviewed in Lazár [24]), we selected the following parameters: i) the maximal quantum yield of PSII photochemistry for a dark-adapted state, Φ_Po_ = (F_M_ - F_0_)/F_M_ = F_V_/F_M_, where F_0_, F_M_, and F_V_ are the minimal, maximal, and variable fluorescence levels, respectively, for a dark-adapted state; ii) the actual quantum yield of PSII photochemistry for a light-adapted state, Φ_P_ = (F_M_’ - F (t))/F_M_’, where F_M_’ and F (t) are the maximal and actual (at time t; usually in the steady state) fluorescence levels for a light-adapted state; iii) the quantum yield of constitutive non-light induced (basal or dark) dissipation processes consisting of Chl fluorescence emission and heat dissipation, Φ_f, D_ = F (t)/F_M_; and iv) the quantum yield of regulatory light-induced heat dissipation, Φ_NPQ_ = F (t)/F_M_’ - F (t)/F_M_. It is worth mentioning here that Φ_P_ + Φ_f, D_ + Φ_NPQ_ = 1; further that Φ_P_ = q_P_Φ_PSII_, where q_P_ (= (F_M_’ - F (t))/(F_M_’ - F_0_’)) is the coefficient of photochemical quenching which estimates a fraction of the so-called open PSII reaction centers; and that Φ_PSII_ (= (F_M_’ - F_0_’)/F_M_’) is the maximal quantum yield of the PSII photochemistry for a light-adapted state. The F_0_’ in the last two equations is the minimal fluorescence level for a light-adapted state which was estimated from: F_0_’ = F_0_/(((F_M_ - F_0_)/F_M_) + (F_0_/F_M_’)) (for details see [24]).

**Figure 5:**
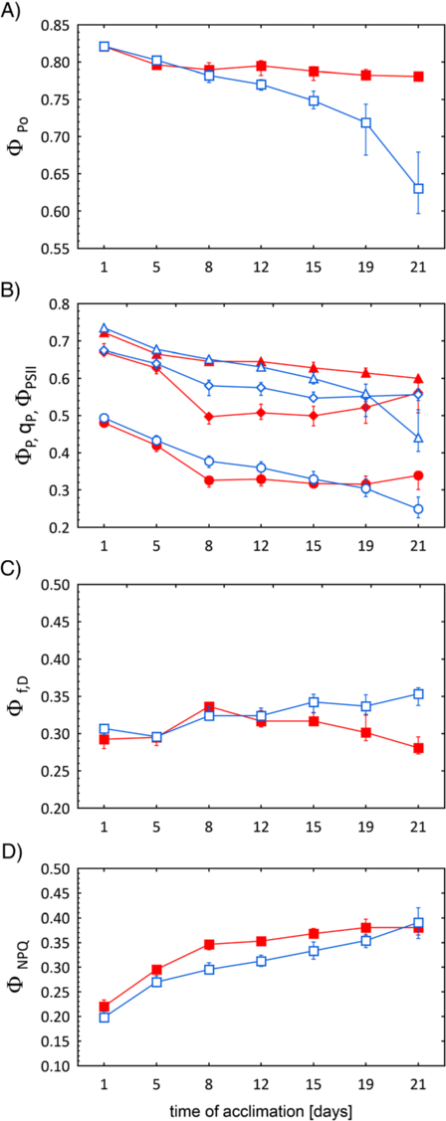
**Changes of CFIN parameters of TER (full symbols) and END (open symbols) pea cultivars measured during the 21 days of cold acclimation.** Changes in **A)** the maximal quantum yield of PSII photochemistry for a dark-adapted state (Φ_Po_); **B)** the maximal and the actual quantum yield of photosystem II photochemistry for a light-adapted state (Φ_PSII_, Φ_P_ respectively), the coefficient of photochemical quenching (q_P_); **C)** the quantum yield of constitutive non-light induced dissipation processes (Φ_f, D_); **D)** the quantum yield of regulatory light-induced heat dissipation (Φ_NPQ_); are shown. The values represent medians from 15 measurements. The error bars represent quartiles. The medians of all the TER and END parameters at the end of measurements were statistically significant (p value < 0.05), except of q_p_ and Φ_NPQ_.

The changes in these Chl fluorescence parameters measured during acclimation of TER and END cultivars to 5°C for 21 days are shown in Figure 5. Φ_Po_ is affected very little by the cold acclimation of TER but there is a continual decrease in Φ_Po_ of END (Figure 5A). Φ_P_ initially decreases more in TER than in END but after 6 days it maintains its value in TER but continues to decrease in END (Figure 5B). The continual decrease in Φ_P_ in END is mostly caused by a continual decrease in Φ_PSII_; q_P_ slightly increasing in the last two measurements in END (Figure 5B). On the other hand, the initial decrease in Φ_P_ in TER is mostly caused by decrease in q_p_ but the almost unchanged value of Φ_P_ in TER after 6 days is caused by the counter action of q_P_, which increases, and of Φ_PSII_, which decreases (Figure 5B). Therefore, it can be concluded that photosynthesis of the two pea cultivars uses different strategies for cold acclimation. Whereas in END, the number of open reaction centers as well as their maximal photosynthetic quantum yield in light generally decrease with prolonged cold acclimation, in TER, a decrease of the maximal quantum yield of PSII photochemistry in light (Φ_PSII_) is compensated by an increase of number of the open PSII reaction centers (q_P_) (Figure 5B). Furthermore, END shows an increased quantum yield of constitutive non-light induced dissipation processes (Φ_f, D_) at the end of the cold acclimation compared to TER (Figure 5C), whereas the rise of the quantum yield of regulatory light-induced heat dissipation (Φ_NPQ_) during the acclimation is faster in TER than in END (Figure 5D).

It is interesting to note that cold-induced changes of the Chl fluorescence parameters for given cultivar and differences (or about the same values) of the parameters between the cultivars (Figure 5) are not accompanied by expected changes and differences of green areas and growth rates (Figure 3). Even when the photosynthetic function was decreased by cold treatment (decrease of the Φ_Po_, Φ_P_, q_P_, and Φ_PSII_ parameters; Figure 5A and 5B), the total and normalized green area of both cultivars was still increased (Figure 3A and 3B). It might show that the grow rate changed (for TER; Figure 3C) or decreased (for END; Figure 3C) with increasing duration of the cold treatment, however, these changes were not statistically significant (data not shown). The uncorrelated behavior of photosynthetic and growth parameters reflects different temperature dependences of photosynthesis and processes hidden behind the plant growth. While photosynthetic function was decreased by treatment of the cultivars at 5°C, probably much lower temperatures would be needed to stop plant growth. Therefore, FCIM data and RGB imaging data carry different and complementary information about acclimation of plants to lower temperatures. To take advantage of the high-throughput capacity of our phenotyping platform, we used a relatively short protocol to measure CFIN. This set up, however, did not allow for determination of photoinactivated centers which might be formed during a joint action of light and cold [25-28]. Depending on the theory used, the formation of the photoinactivated PSII centers can influence all quantum yields of the light-adapted state (for a review see [24]) used in this work. Therefore, in the next study we aim to modify the CFIN measuring protocol in order to determine the quantum yield of photoinactivated PSII centers as well.

**Figure 6:**
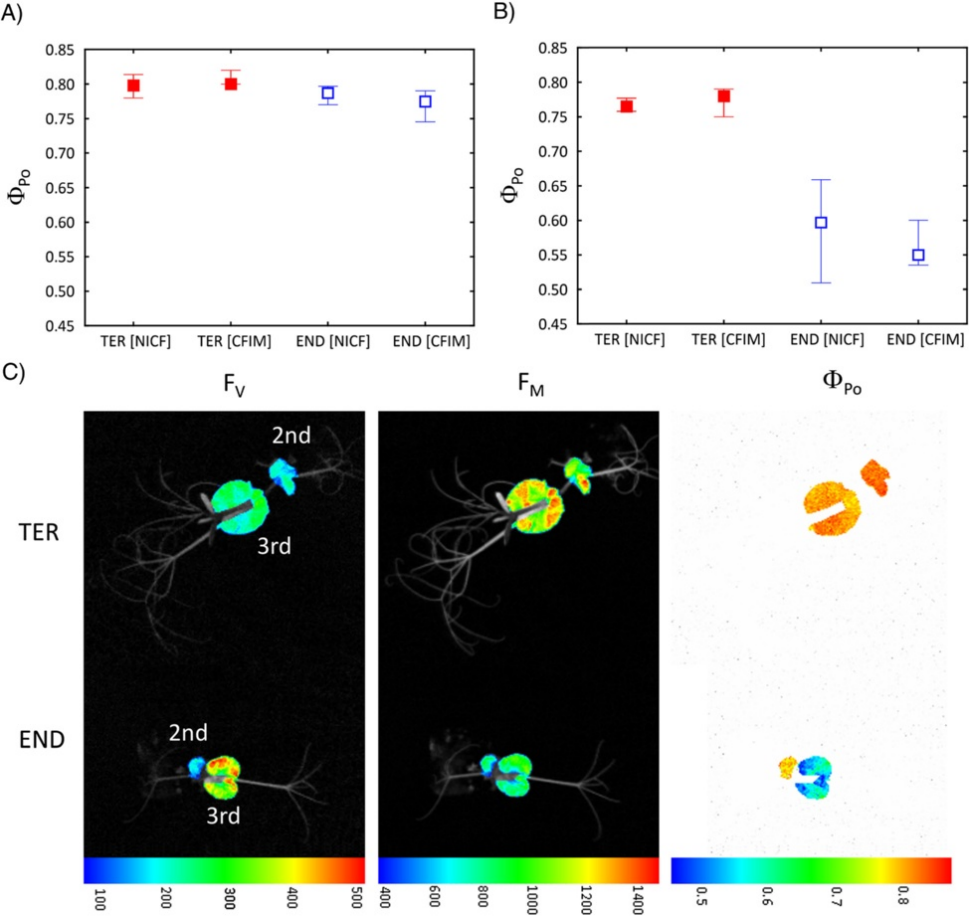
**Comparison of F**_V_ **/F**_M_ **(**Φ_Po_ **) values measured in A) 2nd and B) 3rd leaves by hand-operated non-imaging chlorophyll fluorometer [NICF] or chlorophyll fluorescence imaging [CFIM] part of the platform with manually extracted single leave areas.** The data obtained from each leaf in TER and END genotypes were tested by the Mann–Whitney *U* test showing no significant differences between Φ_Po_ determined by NICF and CFIM (p > 0.05). The values represent medians and the error bars quartiles, respectively. **C)** Imaging of chlorophyll fluorescence in separated leaves of both genotypes. The variable minimal fluorescence (F_V_), maximal fluorescence (F_M_) and maximal quantum yield of PSII photochemistry (Φ_Po_) are shown in false colour scales with relative units.

Furthermore, we tested the reliability and accuracy of the Chl fluorescence parameters measured by the automated CFIM in a high-throughput set up by comparing the selected parameter (Φ_Po_) with the same parameter measured by a hand-operated non-imaging Chl fluorometer. For this purpose the overall Chl fluorescence images were separated into images of the second and third leaves and their Φ_Po_ were evaluated. On the other hand, Φ_Po_ was evaluated from the fast Chl fluorescence rise as measured by the non-imaging Chl fluorometer with a different set of leaves (see Materials and methods). The results of these comparisons are presented in Figure 6A for the second leaves and in Figure 6B for the third leaves, respectively. A representative image of the spatial distribution of Chl fluorescence is presented in Figure 6C. Not surprisingly, the data show that there is no statistically significant difference (at p < 0.05) between Φ_Po_ measured for given leaves by the two different approaches. Moreover, Figure 6C documents another advantage of using the CFIM in automated high-throughput platforms. Although the software is primarily adjusted to calculate the mean value of fluorescence from the total surface of every plant, if needed, the CFIN images can be later separated for subsequent calculation of the Chl fluorescence parameters taken from the individual selected areas which represent individual plant parts (Figure 6C).

To the best of our knowledge, only one study was published reporting on use of CFIM integration into a high-throughput phenotyping platform to analyze cold- or chilling-stress. Using an automated phenotyping platform Jansen et al. [14] evaluated only the F_V_/F_M_ parameter (Φ_Po_) for two different *Arabidopsis* plants (wild-type and a mutant), and wild-type tobacco plants. Φ_Po_ decreased in the wild-type tobacco plants during the cold treatment, and the same decreasing trends were found with *Arabidopsis* plants, however, the differences between the wild-type and a mutant were not convincing. Using a CFIM system, Lootens et al. and Devacht et al. [25,29] studied the effect of different cold temperatures on industrial chicory plants. In agreement with our results, the authors found again only a small decrease of Φ_Po_ after 10-day incubation at 4°C and the values of the Φ_P_ and Φ_PSII_ parameters caused by the incubation were similar to those obtained in our study. Mishra et al. [30,31] used CFIM to study the effect of a two-week incubation at 4°C on nine *Arabidopsis thaliana* accessions differing in cold tolerance. In addition to evaluation of standard Chl fluorescence parameters, like Φ_Po_, Φ_P_, and q_P_, the authors also showed that combinatorial imaging of Chl fluorescence transients combined with classifier and feature selection methods could discriminate between detached leaves from cold sensitive and cold tolerant accessions.

## Materials and methods

### Plant material

Two morphologically similar field pea (*P. sativum* subsp. *sativum* var. *sativum*) cultivars Terno (TER) and Enduro (END) were used in the experiment. TER is pea cultivar, used for spring sowing term with a certain capacity to cold-acclimation, whereas END is a cold-tolerant overwintering cultivar. The END cultivar was obtained from the Selgen a.s. company (Prague, Czech Republic). The TER cultivar was taken from the Czech collection of pea genetic resources kept in Agritec Ltd., Šumperk, Czech Republic. The collection is run according to the general rules of the National Programme for Plant Genetic Resources of the Czech Republic and the passport data are available on http://genbank.vurv.cz/genetic/resources/.

### Cultivation conditions and experimental setup

The TER and END pea cultivars were sown into standardized pots (65 x 65 x 95 mm, Plant-It-Rite, Australia) filled with 100 g of soil (Substrate 2, Klasmann-Deilmann GmbH, Germany) and watered to full water capacity. The seeds were germinated in mini-greenhouses (50 x 32 x 6 cm with clear plastic lid) in a growth chamber with white LED lighting (150 μmol photons of PAR m^-2^ s^-1^). The conditions were set-up to simulate a long day (16 h day, 8 h night) with temperatures of 22°C during the light period and 20°C in the night. The relative humidity was set to 60%. After the development of the first true leaves, the temperature was decreased to 5°C for the entire experiment, the other parameters remained unchanged. The plants were regularly watered with the same amount of water. Fifteen seedlings from each cultivar were used for the automated phenotyping, and another fifteen plants were used for control measurements of maximal quantum yield of PSII photochemistry through the use of a hand-operated non-imaging Chl fluorometer. For measurements in PlantScreen^TM^ phenotyping platform (Photon Systems Instruments, Brno, Czech Republic), the pots with the seedlings were placed in standardized trays; two pots per tray and automatically loaded and measured by the platform. The movement of the trays was performed by a robotic-driven conveyor belt that routinely transferred experimental plants between the growing and measuring areas according to a user-defined protocol. A single measuring round of 8 trays consisted of 20 minutes of dark-adaptation, followed by the measurement of Chl fluorescence and digital RGB imaging from three optical projections. Approximately 16 plants per hour were analyzed, due to the length of the measuring round that is dependent on the length of the dark adaptation and CFIM measurement. In the case of RGB imaging the platform throughput increases to about 60 experimental trays (120 plants) per hour. The data from Chl fluorescence and RGB imaging were stored in a database server, and analyzed either by the software provided by the manufacturer or by the software developed by the authors of this study as described below.

### RGB software image analysis

The plants were automatically loaded into the measuring cabinets of the PlantScreen^TM^ platform where the three RGB images – the top, front, and side views – (Figure 1) of each experimental tray containing two plants were taken. To assess the total green area, the green mask of the individual plants has to be found in the image. To this end, we used a combination of automatic thresholding procedures and automatic edge detection techniques. First, the image was converted from the RGB colour space into the HSV colour space. It is much easier to find the green mask in the H channel of the HSV colour space because the S and V channels only contain information on the saturation and brightness of the colour but not the hue itself. The region in the three dimensional RGB space which defines the ‘plant green’ colour may have a rather complicated shape, however, it is reduced to a line-segment in the one-dimensional H space as the S and V coordinates can be ignored. For thresholding in the H channel, several standard automatic algorithms can be used, e.g., the most popular Otsu method [32] that calculates the optimum threshold separating the foreground and background pixels so that their combined intra-class variance is minimal. In our case, we used an even simpler technique - foreground (i.e., the plant) was predefined as a particular line segment in the H channel. This was possible due to the standardized image acquisition setting.

The thresholding step usually provides very good discrimination between the plant and its background and no further processing is necessary. However, the pea plants possess very thin offshoots (only one or two pixels thick) that may be difficult to find by thresholding alone. If the thresholding routine makes a single-pixel mistake, which often happens due to noise in the image, the entire offshoot is lost, which is undesirable. We solved this problem by exploiting the Canny automatic edge detection algorithm which tracks the contours of the plant image [33]. The thin offshoots were tracked particularly well because the edge detection algorithm focused on such thin structures. The results of the thresholding step were then combined with the edge detection step and the final green mask of the object was found. Finally, a couple of post processing steps were performed (e.g. median filtering and image opening and/or closing) to enhance the quality of the mask.

It only took several seconds on a standard PC to find the green mask of a single pea plant. The mask provided information about the projection of the plant surface area onto the three image planes. The projections can be expressed in square millimeters because the RGB camera had been calibrated beforehand. The calibration proceeded as follows. Two bars covered by millimeter paper were placed in the pots instead of the pea plants. The bars were approximately the same height as the plants. Three images (top, front, side) of the two bars were acquired with the same camera setting used for the entire experiment. These images served as the standard for converting the leaf area from pixels to square millimeters. The total green area of the plant is then estimated as A = √(A_x_^2^ + A_y_^2^ + A_z_^2^), where A_x_, A_y_, and A_z_ are the respective projections onto the three image planes. This procedure is naturally not precise but it gives an estimate which is in good correlation (Figure 4) with the fresh biomass of the above ground plant parts.

### CFIM and non-imaging Chl fluorescence measurements

A standard protocol was used for the measurement of Chl fluorescence quenching using the CFIM part of the PlantScreen^TM^ platform. The plants underwent 20 – 40 minutes of dark adaptation before CFIM measurements. During all signal recordings, short (33.3 μs) red (650 nm) “measuring” flashes were applied and a Chl fluorescence signal was detected a few microseconds before the measuring flash and during the flash, and then the two signals were subtracted. This is a pulse amplitude modulation (PAM) type of measurement. To measure the minimal fluorescence for a dark-adapted state, F_0_, only the measuring flashes were applied for an initial 5 seconds. Then, a saturation pulse of 800 ms duration (white light, intensity of 1000 μmol photons of PAR m^-2^ s^-1^) was applied and the maximal fluorescence for a dark-adapted state, F_M_, was measured. After the F_M_ measurement, fluorescence was kept relaxed in darkness for 17 seconds. Red actinic light (650 nm, intensity of 100 μmol photons m^-2^ s^-1^) was then switched on for 70 seconds to drive photosynthesis. It was visually checked so that a steady state fluorescence signal was attained at 70 s of illumination. During the actinic illumination, saturation pulses were applied at 8, 18, 28, 48, and 68 seconds from the beginning of the actinic illumination. The value of the maximal fluorescence measured during the last saturation pulse was taken as the maximal fluorescence signal for the light-adapted state, F_M_’. The fluorescence signal caused by the actinic illumination measured just before the last saturation pulse was taken as the steady state fluorescence for a light-adapted state, F (t). The four fluorescence levels (F_0_, F_M_, F (t), F_M_’) were used for calculation of the minimal fluorescence level for a light-adapted state, F_0_’, the quantum yields, and the other fluorescence parameters as defined and described in the Results section.

A hand-operated FluorPen fluorometer (Photon Systems Instruments, Brno, Czech Republic) was used for control measurements in order to compare the results obtained using automatized CFIM with hand-operated non-imaging Chl fluorescence measurements. Blue light (455 nm) of intensity 1000 μmol photons m^-2^ s^-1^ and a duration of 1 second was used by FluorPen for illumination of the sample and a whole fast fluorescence rise (the O-J-I-P curve) was recorded. However, only the minimal and maximal fluorescence levels, F_0_ and F_M_, respectively, for the dark adapted state, were evaluated from the curve using built-in routines. The two fluorescence levels were used for calculation of the maximal quantum yield of PSII photochemistry (see Results). The data for Chl fluorescence measurements are presented as medians and lower and upper quartiles [34].

## Conclusion

In this proof-of-concept study, the high-throughput method for automated screening of cold-tolerant pea (*Pisum sativum* L.) cultivars was designed. TER and END cultivars were screened simultaneously in an automated way with throughput of 16 plants per hour for i) growth of the aerial parts by RGB imaging and ii) for the efficiency of photosynthesis by chlorophyll fluorescence imaging. We demonstrated that the presented integrative approach based on analyses of differences in relative growth rate and selected CFIM parameters can provide deeper insight into the physiological base of cold-acclimation. Data from both analytical tools pointed to significant differences in the growth and photosynthesis of TER and END cultivars, and indicated that the two pea cultivars use different strategies for cold acclimation differing in number of open PSII reaction centers, their maximal photosynthetic quantum yield in light and quantum yield of constitutive non-light induced dissipation processes. The reliability of the screening was verified by independent measuring of the fresh weight of the shoots and by Chl fluorescence measurement by hand fluorometer. Since the CFIM analysis is not limited to plant morphology and our image analysis was sensitive enough to detect tiny tendrils of pea, we believe that the described procedure can be easily employed for shoot analyses of other different plant species.

## Abbreviations

Chl: Chlorophyll
CFIM: Chlorophyll fluorescence imaging
CFIN: Chlorophyll fluorescence induction
END: Enduro
F_0_ and F_0_^’^: Minimal chlorophyll fluorescence levels for dark- and light-adapted states, respectively
F_M_ and F_M_^’^: Maximal chlorophyll fluorescence levels for dark- and light-adapted states, respectively
FW: Fresh weight
F (t): Actual (at time t; usually in the steady state) fluorescence level for light-adapted state
F_V_: Variable chlorophyll fluorescence level for a dark-adapted state
Φ_Po_: The maximal quantum yield of photosystem II photochemistry for a dark-adapted state
Φ_P_: The actual quantum yield of photosystem II photochemistry for a light-adapted state
Φ_PSII_: The maximal quantum yield of photosystem II photochemistry for a light-adapted state
Φ_f, D_: The quantum yield of constitutive non-light induced (basal or dark) dissipation processes consisting of fluorescence emission and heat dissipation, Φ_NPQ_, The quantum yield of regulatory light-induced heat dissipation
GA: Green area
NICF: Non-imaging chlorophyll fluorescence fluorometer
NGA: Normalized green area
RGB: Red-green-blue
RGR: Relative growth rate
PAM: Pulse amplitude modulation
PAR: Photosynthetic active radiation
PSII: Photosystem II
q_P_: The coefficient of photochemical quenching
TER: Terno
